# Matching the genetics of released and local *Aedes aegypti* populations is critical to assure *Wolbachia* invasion

**DOI:** 10.1371/journal.pntd.0007023

**Published:** 2019-01-08

**Authors:** Gabriela de Azambuja Garcia, Gabriel Sylvestre, Raquel Aguiar, Guilherme Borges da Costa, Ademir Jesus Martins, José Bento Pereira Lima, Martha T. Petersen, Ricardo Lourenço-de-Oliveira, Marion F. Shadbolt, Gordana Rašić, Ary A. Hoffmann, Daniel A. M. Villela, Fernando B. S. Dias, Yi Dong, Scott L. O’Neill, Luciano A. Moreira, Rafael Maciel-de-Freitas

**Affiliations:** 1 Laboratório de Mosquitos Transmissores de Hematozoários, Instituto Oswaldo Cruz, Fiocruz, Rio de Janeiro, Brazil; 2 Serviço de Jornalismo e Comunicação, Instituto Oswaldo Cruz, Fiocruz, Rio de Janeiro, Brazil; 3 World Mosquito Program, Fiocruz, Rio de Janeiro, Brazil; 4 Laboratório de Fisiologia e Controle de Artrópodes Vetores, Instituto Oswaldo Cruz, Fiocruz, Rio de Janeiro, Brazil; 5 Instituto Nacional de Ciência e Tecnologia em Entomologia Molecular (INCT-EM)/CNPq, Rio de Janeiro, Brazil; 6 School of BioSciences, Bio21 Institute, University of Melbourne, Parkville, Victoria, Australia; 7 Programa de Computação Científica, Fiocruz, Rio de Janeiro, Brazil; 8 Gabinete da Presidência, Fiocruz, Rio de Janeiro, Brazil; 9 Institute of Vector-Borne Disease, Monash University, Clayton, Victoria, Australia; 10 Instituto de Pesquisas René Rachou, Belo Horizonte, Fiocruz, Minas Gerais, Brazil; Faculty of Science, Mahidol University, THAILAND

## Abstract

**Background:**

Traditional vector control approaches such as source reduction and insecticide spraying have limited effect on reducing *Aedes aegypti* population. The endosymbiont *Wolbachia* is pointed as a promising tool to mitigate arbovirus transmission and has been deployed worldwide. Models predict a rapid increase on the frequency of *Wolbachia*-positive *Ae. aegypti* mosquitoes in local settings, supported by cytoplasmic incompatibility (CI) and high maternal transmission rate associated with the *w*MelBr strain.

**Methodology/principle findings:**

*Wolbachia w*MelBr strain was released for 20 consecutive weeks after receiving >87% approval of householders of the isolated community of Tubiacanga, Rio de Janeiro. *w*MelBr frequency plateued~40% during weeks 7–19, peaked 65% but dropped as releases stopped. A high (97.56%) maternal transmission was observed. Doubling releases and deploying mosquitoes with large wing length and low laboratory mortality produced no detectable effects on invasion trend. By investigating the lab colony maintenance procedures backwardly, pyrethroid resistant genotypes in *w*MelBr decreased from 68% to 3.5% after 17 generations. Therefore, we initially released susceptible mosquitoes in a local population highly resistant to pyrethroids which, associated with the over use of insecticides by householders, ended jeopardizing *Wolbachia* invasion. A new strain (*w*MelRio) was produced after backcrossing *w*MelBr females with males from field to introduce mostly pyrethroid resistance alleles. The new strain increased mosquito survival but produced relevant negative effects on *Ae*. *aegypti* fecundity traits, reducing egg clutche size and egg hatch. Despite the cost on fitness, *w*MelRio successful established where *w*MelBr failed, revealing that matching the local population genetics, especially insecticide resistance background, is critical to achieve invasion.

**Conclusions/significance:**

Local householders support was constantly high, reaching 90% backing on the second release (*w*MelRio strain). Notwithstanding the drought summer, the harsh temperature recorded (daily average above 30°C) did not seem to affect the expression of maternal transmission of *w*Mel on a Brazilian background. *Wolbachia* deployment should match the insecticide resistance profile of the wild population to achieve invasion. Considering pyrethroid-resistance is a widely distributed phenotype in natural *Ae*. *aegypti* populations, future *Wolbachia* deployments must pay special attention in maintaining insecticide resistance in lab colonies for releases.

## Author summary

Traditional vector control methods have been showing limited success in managing arboviruses disease outbreaks in endemic areas. The endosymbiont bacterium *Wolbachia* was microinjected on the mosquito *Aedes aegypti* and later showed the ability to block dengue, chikungunya and Zika viruses’ development on vector body. Nowadays, *Wolbachia*-infected *Ae*. *aegypti* has been deployed in twelve countries as an attempt to mitigate arboviruses transmission. Herein, we report the failure of *Wolbachia* releases on a suburban isolated district of Rio de Janeiro/Brazil. For the first time, we demonstrated that the insecticide resistance profile of released and wild mosquitoes is a critical feature for achieving *Wolbachia* invasion. Special consideration must be given to avoid releasing susceptible mosquitoes where native populations are resistant, since local householders might rely on domestic use of spraying insecticides (mostly pyrethroids) to protect themselves from mosquito bites.

## Introduction

Mosquito-borne diseases heavily impact human health in tropical areas, where some of them co-circulates in several countries. Dengue virus (DENV) is a flavivirus distributed mainly in tropical and subtropical areas, causing around 400 million new infections each year. Brazil has the highest number of DENV cases, with more than one million cases per year for the last three years [1]. Chikungunya virus (CHIKV) was added to the arboviruses of global health concern in the early 2000‘s, when a new epidemic strain emerged from an enzoonotic lineage. In 2014, the first autochthonous CHIKV cases appeared in Brazil, linked to both the Asian and the East Central South Africa (ECSA) genotypes of the virus, with the last spreading throughout the country [2]. Most recently, the world witnessed the spread of Zika Virus (ZIKV) across the Western Hemisphere within the span of one year. In 2014, ZIKV was introduced in Brazil from the Pacific Islands, and one year later a spike in microcephaly cases among newborn babies prompted the World Health Organization (WHO) to declare a Public Health Emergency of International Concern [3]. Since late 2016, one of the most severe sylvatic yellow fever outbreak has been reported on the gates of the largest metropolitan Brazilian areas highly infested with *Aedes aegypti*, augmenting the risk of urban outbreaks of YF [4].

All aforementioned arboviruses are or may be transmitted by the mosquito *Ae*. *aegypti*, making the control of mosquito populations the best action to mitigate disease transmission. Existing vector control programs in Brazil rely heavily on the use of insecticides and reduction of larval breeding sources to limit vector abundance. However, due to widespread insecticide resistance in wild *Ae*. *aegypti* populations and the logistical constraints in extending source reduction over large cities, the effectiveness of traditional control methods in this context is limited [5–7]. Therefore, the development of new strategies to supplement traditional vector control methods is of utmost importance to manage mosquito-borne diseases [8].

One of the innovative approaches consists of deploying the endosymbiotic bacterium *Wolbachia pipientis* to block arbovirus transmission in endemic urban settlements [9–13]. *Wolbachia* are maternally transmitted bacteria present in around 60% of all arthropod species. Although they do not naturally infect *Ae*. *aegypti*, a few strains were transinfected into *Ae*. *aegypti* from other insects and their pathogen-blocking properties were discovered [9–15]. *Wolbachia* spread through arthropod populations based on its maternal mode of transmission and reproductive manipulations to the host, known as cytoplasmic incompatibility. Using these properties, *Wolbachia-*infected *Ae*. *aegypti* can be released in disease-endemic areas to replace a local mosquito population of high vector competence with others with diminished potential for transmission.

Successful releases of *Wolbachia* strain *w*Mel have been carried out in central Vietnam and Northern Queensland [13]. Given these promising pilot releases and the limited effect of insecticide-based control campaigns [7], the Brazilian Ministry of Health and the World Mosquito Program (formerly Eliminate Dengue) pursued rolling out *w*Mel strain in the community of Tubiacanga, Rio de Janeiro. After releasing a total of 180,000 *Ae*. *aegypti* carrying *w*MelBr (Brazilian strain) during 20 consecutive weeks, we observed that *Wolbachia* was unable to spread into the *Ae*. *aegypti* population. After exhaustive investigation, we herein report the factors that jeopardized the *Wolbachia* invasion, the steps required to produce a fit mosquito strain, and the results of a subsequent release on the same site.

## Materials and methods

### Study sites

Mosquitoes infected with the *Wolbachia* strain *w*Mel were released in the isolated community of Tubiacanga (22°47'06"S; 43°13'32"W) in Rio de Janeiro. This is a lower-middle-class community located in a lowland coastal area on the shores of Guanabara Bay comprised of around 3,000 residents in 690 houses [16,17]. During releases, weekly climatic data (mean, minimum and maximum temperature, accumulated rainfall) was collected at a meteorological station located 6 km from Tubiacanga (S1 Fig).

### Ethical considerations

*Wolbachia* releases were authorized by the National Research Ethics Committee (CONEP, CAAE 02524513.0.1001.0008), and further regulatory approval was obtained by three Government agencies: IBAMA (Ministry of Environment), Anvisa (Ministry of Health) and MAPA (Ministry of Agriculture). According to ethical and regulatory approval, door-to-door activity and formal signed consent for the releases was required from at least 70% of the sampled households (around 30% of households were sampled). Adults over 21 years old answered the questionnaires. One strong request of CONEP was that the World Mosquito Program team not change any practices performed by the community or the municipality vector control.

The maintenance of *Ae*. *aegypti* colonies in the lab were achieved by blood feeding mosquitoes with human blood obtained from anonymous donors from the blood bank of the Rio de Janeiro State University, whose blood bags would be discarded due to small volume. We have no information on donors, including sex, age and clinical condition, but the blood bank discard those bags positive for Hepatitis B, Hepatitis C, Chagas disease, syphilis, HIV and HTLV. Before offering the blood for mosquitoes, it was screened for DENV using the Dengue NS1 Ag STRIP (Bio-Rad). The use of human blood was approved by the Fiocruz Ethical Committee (CAAE 53419815.9.0000.5248).

### Community engagement (CE) and communication (Comms) strategy for *Wolbachia* release

Before any mosquito releases, community activities were carried out with the purpose of presenting the project, listening to the population, explaining the implications for the community as well as for individuals. CE began with the identification of local stakeholders, in a process of engagement with the community as the initial stage of an action-participant research [18,19].

In order for community bonds to be strengthened and a process of governance and scientific transparency to be established, a Community Reference Group (CRG) was created. The CRG had seven local volunteered representatives (two from the community association, one from local school, one health agent, one merchant and two residents). This stage describes the process of defining the problem and planning of actions [20]. The CRG was built as a space for dialogue between the parties, with shared advances and concerns regarding public health issues, especially those related to vector-borne diseases, and the safety related to *Wolbachia* releases. The meetings happened monthly and were held, as a rule, at the local residents' association. During the engagement and communication process, lectures were given in schools, training in the local health unit, participation in meetings of local social groups, and door-to-door actions to explain the project. A survey, conducted by an external team, evaluated the consent of local residents supporting releases.

With the need for a second round of mosquito release (*w*MelRio), new Community Engagement and Communication strategies were developed. Firstly, we focused on our closest partners, the residents’ association and the CRG. We explained the reasons for *Wolbachia* not invading, listened to community expectations and discussed the timeline for the second release. The door-to-door activities were intensified, meetings with householders were increased and we also strengthened the collaboration with the local primary school. The Communications team produced flyers to support CE activities, placed banners in public areas, scheduled door-to-door visits whenever required and provided positive mass media on TV, radio and internet. After these actions, another survey was carried out.

### Backcrossing of *w*MelBr-infected *Ae*. *Aegypti*

*w*Mel-infected *Ae*. *aegypti* were imported from Australia (IBAMA license 11BR005873/DF) to produce a *Wolbachia*-infected line with a Brazilian genetic background, named *w*MelBr (details on [17]). The backcrossed laboratory colony was maintained for 17 generations before the obtaining regulatory approvals for mosquito releases. Subsequently, every five generations we added 10% of wild males from a Rio de Janeiro population mix to refresh the genetic pool of the colony.

### *w*MelBr colony quality assurance

We randomly selected 100 3^rd^ -4^th^ instar larvae per generation to estimate the frequency of *Wolbachia* in the colony, with a threshold of at least 98% positivity to support releases. Larvae were reared with 0.45 g of Tetramin Flakes (Tetra GmbH, Herrenteich, Germany) every two days in 3 L plastic trays at 28°C. Each week ten of the release cups (50 mosquitoes per cup) were brought back to the lab to assess the fitness of mosquitoes being released. We assessed male and female wing size, adult mortality, and sex ratio. Wing length was defined as the distance from the axillary incision to the apical margin excluding the fringe [21]. Mortality and sex ratio were checked visually as release cups returned to the insectary, when mosquitoes were one week old.

### *w*MelBr and *w*MelRio releases

The first release started four months after regulatory approval, i.e., 17 generations had elapsed with limited outbreeding (adding 10% wild males every five generations). In the first round of releases at Tubiacanga, 50 mosquitoes 4–6 days old were released in the early morning (5:00 AM) in front of one in every four houses. The deployment of *w*MelBr began in September 2014 and lasted 20 consecutive weeks, until January 2015. The total number of mosquitoes released per week increased over time, starting with 7,500 on week 1 and ending with 15,000 on week 20. Therefore, we released between 10.86 to 21.74 mosquitoes per house per week. Later, on August 2015, we started deploying *w*MelRio in Tubiacanga. A similar release protocol was adopted, with 10,000 mosquitoes released every week through 24 consecutive weeks.

### *Wolbachia* detection

Adult mosquitoes were individually screened for the presence of *Wolbachia* infection using a multiplex RT-qPCR that included the WD0513 gene and a ribosomal gene of *Ae*. *aegypti* [22]. The amplification was carried out on a ViiA-7 Machine (Thermo Scientific) using Taqman Universal PCR Master Mix (Thermo Scientific) following manufacturer’s instructions. *Wolbachia* relative quantification was performed according with Caragata et al. [23].

### Mitochondrial DNA

To test for imperfect maternal transmission of *Wolbachia* in released mosquitoes, we conducted the analysis of mitochondrial DNA variation on field-caught females. We screened for a diagnostic SNP within the mitochondrial *COXI* gene in mosquitoes that were negative for *Wolbachia*. This SNP is located at the 531bp location in the *COI* amplicon (A in release strain mosquitoes, and G in wild type mosquitoes). As *Wolbachia* and mitochondria are coinherited we can infer that individuals that do not contain *Wolbachia* but carry the diagnostic SNP have arisen from imperfect transmission of *Wolbachia* [24]. Mosquitoes were trapped 1, 3, 12, 13, 14, 16, 17, 20 and 26 weeks after the release of *w*MelBr mosquitoes had started. Those identified as *Ae*. *aegypti* were tested for the *Wolbachia* infection status by multiplex qPCR using Taqman probes except the primer set IS5 were not used in this assay. Presence of the diagnostic SNP in the COXI gene was tested in 456 mosquitoes using the Sanger sequencing of a small fragment (970bp) of the *COXI* mitochondrial gene [24]. Sequence identity was validated using BLASTn at NCBI, and the alignment was performed using SeqMan Pro (Lasergene, Madison, USA).

### *w*MelRio backcrossing

When the need for releasing mosquitoes resistant to pyrethroids into field populations became apparent, we started a second backcrossing using 500 females from *w*MelBr colony and 500 males from Urca, an urban district from Rio with high pyrethroid resistance. To infer pyrethroids resistance throughout backcrossing, we analyzed *kdr* mutations frequencies. These mutations have been described as an important insecticide resistance mechanism that contributes with high levels of pyrethroid resistance in Rio de Janeiro *Ae*. *aegypti* populations [25–27]. Moreover, *kdr* scoring is a rapid and simple tool when compared with bioassays. During backcrossing, we had a total of six populations, each one representing one step of backcrossing to produce *w*MelRio (*w*MelBr, Field (Urca), *w*MelF1CP, *w*MelB1, *w*MelB2, and then *w*MelRio). We added a seventh population, *w*MelBrTet, which was treated with tetracycline by three successive generations to remove *Wolbachia* and maintain the mosquito genetic background [17] (S2 Fig). Doing so, we expected that the frequency of *kdr* alleles increased over time, what was tested by *Wolbachia* and *kdr* screening of 100 mosquitoes randomly sampled for each generation.

### Monitoring *kdr* frequency during and after releases

Wild *Ae*. *aegypti* populations in Brazil contain two *kdr* alleles: substitution restricted to the 1534 position (Na_V_R1) or concurrent substitutions in both 1534 and 1016 sites (Na_V_R2) [25–27]. These alleles are found at different frequencies and in the Na_V_R2 homozygous state pose a severe fitness cost [28]. We estimated the frequency of *kdr* alleles (Na_V_R1 and Na_V_R2) as a proxy for pyrethroid resistance in Tubiacanga and released mosquitoes using a customized Taqman genotyping assay (ThermoFischer Scientific). Genotypic and allelic frequencies were assessed at 1016 and 1534 sites: Na_V_S (1016 Val^+^ + 1534 Phe^+^), Na_V_R1 (1016 Val^+^ + 1534 Cys^*kdr*^) and Na_V_R2 (1016 Ile^*kdr*^ + 1534 Cys^*kdr*^). On weeks 1, 8, 16, 20 (during *w*MelBr release), 26, 30, 34 (between releases), 51, 58, 66, 70 (during *w*MelRio release) and 76, 80 and 84 (after *w*MelRio release), we randomly selected at least 60 field-caught *Ae*. *aegypti* females per week captured on BG-Sentinel traps, among which 30 were positive for *Wolbachia* and 30 were negative, i.e., wild-type females collected within release site (according to qPCR screening).

### Insecticide resistance bioassays in field and released mosquitoes

To determine the insecticide resistance profile, we performed bioassays on *w*MelBr, *w*MelRio, and field mosquitoes from Tubiacanga, Jurujuba and Urca. The susceptible Rockefeller strain was used as a control. Dose-response bioassays were performed with third instar larvae to determine their susceptibility to the organophosphate temephos (Pestanal, Sigma-Aldrich) and the chitin synthesis inhibitor diflubenzuron, and with 3-5-day-old adult females to the pyrethroid deltamethrin and the organophosphate malathion using WHO test tubes [29,30]. Lethal concentrations were calculated with Probit analysis [software Polo-PC, LeOra Software, Berkeley, CA] [31]. Resistance Ratios (RR_50_ and RR_90_) were obtained by dividing the lethal concentrations (LC) or *knockdown* time (*Kd*T) values of the test populations by those of the Rockefeller reference strain [29,30].

### Estimating fitness costs of pyrethroid resistance in the laboratory colony

The frequencies of the *kdr* allele were obtained from a sample of 100 mosquitoes of F1 and F18 generations from the *w*MelBr colony. Wild males were added every five generations to refresh the colony, and consequently resistance alleles were introduced. We estimated the fitness cost due to insecticide resistance by applying a Monte-Carlo procedure over a two-allele model. A fitness cost applies for the genotype of homozygous resistant alleles (R1R1 and R2R2, similarly), whereas heterozygous individuals have a smaller cost given by a multiplicative factor of 0.9 as used by Hoffmann and Turelli [32] in the case of resistance nearly recessive. Successive *w*MelBr crossings were simulated for 17 generations assuming these fitness costs. In generations F10 and F15 we also simulated crossings with an added male population of resistant homozygous individuals by a ratio of 1 male: 10 females. After running 100,000 simulations, we obtained a subset of results for which the predicted frequencies in F18 were equal to the observed frequency for the F18 generation ±1%.

### Fitness assays

Mosquito life-history traits were measured in the backcrossed strain to test whether the addition of alleles to promote pyrethroid resistance would affect the fitness of *Wolbachia*-infected mosquitoes (S2 Fig).

#### Development time

Eggs of the seven tested populations were hatched, and three groups of 100 first instar larvae per population were transferred to plastic trays (33 X 24 X 8 cm) and monitored daily for 14 days to assess the number of pupae per day. Survival analysis was performed to determine the time to pupation in the populations.

#### Adult survival

Sixty females per population were selected and individually isolated in labeled cylindrical plastic vials (6.5 cm height, 2.5 cm diameter). Adults were maintained under insectary conditions (80 ± 5% humidity and 25 ± 3°C) and fed ad libitum with 10% sucrose solution for up to 36h before the blood feeding [33]. Survival was checked daily and dead mosquitoes were stored in a -80°C freezer for subsequent *Wolbachia* and *kdr* detection. *Ae*. *aegypti* longevity was normally distributed (Shapiro-Wilk W = 0.9942, P = 0.113). The effect of *Wolbachia* infection and density, frequency of *kdr* mutation and its interaction on mean *Ae*. *aegypti* longevity was analyzed by a three-way ANOVA. A Log-rank test followed by Bonferroni correction compared the survival distribution of *Ae*. *aegypti* females. We estimated by Cox proportional risk the hazard ratio of *Wolbachia* density and *kdr* frequency on *Ae*. *aegypti* survival.

#### Oviposition success, female fecundity and egg hatch

Females were blood fed with human blood once a week and eggs were counted 3–4 days later. Fecundity was analyzed by considering the first five clutches of eggs laid, since only a small number of females laid eggs beyond the fifth clutch, precluding adequate numbers for analysis. We analyzed the likelihood that a mosquito laid at least one egg (at a given clutch) with a logistic analysis that included *Wolbachia* presence and density, frequency of *kdr* mutation and clutch-number (i.e., age), backwards-eliminating the non-significant interactions. Second, we analyzed the number of eggs of the successful mosquitoes with a MANOVA repeated measures analysis. We included clutch-number as the repeat and estimated the effects of *Wolbachia* infection, *Wolbachia* density and frequency of *kdr* mutation.

Eggs laid on filter papers were kept for four days at room temperature to allow for embryo development. Eggs were counted and the batch was hatched in plastic cups with Tetramin as a food resource. Larvae were counted and divided by the number of eggs to assess mosquito fertility. Egg hatch rate was monitored during the four first oviposition cycles.

#### Maternal transmission

All adult females and a sub-sample of up to 20 larvae per female were screened for *Wolbachia* on weeks 1 and 4. To assess whether there was any failure of maternal transmission and, if present, to test if it was correlated with the introduction of *kdr* during backcrossing.

Statistical analysis was conducted on R version 3.3.2 and JMP 13.

### Genetic similarity among release and field mosquitoes

To assess the level of genetic similarity between released material and wild mosquitoes, we used double-digest RAD-sequencing, as previously described [34]. We analyzed 25 mosquitoes from each sample: the released colonies (*w*MelBr and *w*MelRio), field-caught individuals from Tubiacanga and Gordonvale (Queensland, Australia, as a proxy for the original *w*Mel transfected colony). The 100 bp paired-end reads from the Illumina HiSeq platform were deposited to NCBI (SRA PRJNA273913, PRJNA330553). The raw sequences were processed through a previously published pipeline [34], retaining high-quality reads (Phred >20), trimmed to 90 bp, and uniquely aligned to the *Ae*. *aegypti* genome sequence (AaegL1) using Bowtie v0.12.7 [35]. SNPs and genotypes were called in the program Stacks v1.36 [36], and further filtering of markers was done in the program VCFTools v0.1.12b [37]. Genome-wide similarity among samples was analyzed using the Discriminant Analysis of Principal components [38] in the R package adegenet [39]. The index of genetic structuring (*F*_ST_) was calculated in VCFTools using the Weir and Cockerham’s AMOVA-based method [40].

## Results

### Community approval

An action-participant research requires a qualitative-quantitative analysis, thus, throughout the process of engagement and communication, some indicators sustained the success of the adopted actions. The CRG summing the willingness of local institutions to develop project-related activities, indicated that the acceptance of the community, since the proposed activities were not mandatory and there was no resistance from the actors involved. Moreover, it has identified a growing involvement of the community, willing to act cooperatively and actively in the territory. In this process, a sense of belonging was identified. In informal communications, we identified that the locals were proud to be the first Latin America neighborhood to participate in the project and receive a *Wolbachia* release, bringing a positive agenda for that community. A house-to-house survey was conducted in 268 (38.8%) houses in Tubiacanga in June/2014. A total of 235 (87.7%) householders declared support to mosquito releases (*w*MelBr strain), 20 (7.4%) opposed releases and 13 (4.85%) did not answer this specific question. A new survey was required after the second trial was confirmed, and the approval was over 90% in Tubiacanga, demonstrating long-term and strong community support for *Wolbachia* deployment.

### *w*MelBr deployment in Tubiacanga

**Fig 1:**
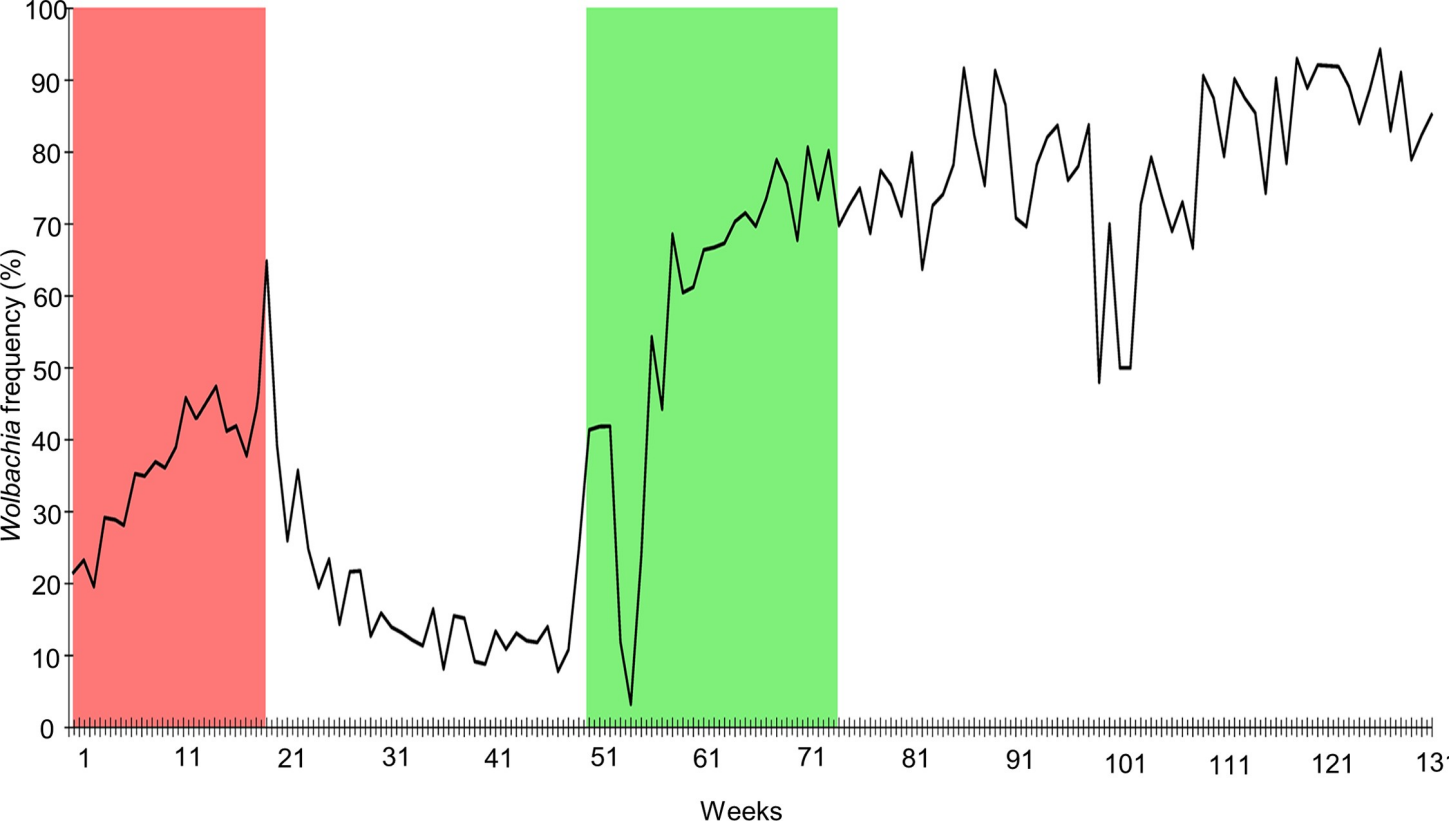
The frequency of *w*MelBr and *w*MelRio strains during *Wolbachia* deployment in Tubiacanga. Red area represents the *w*MelBr releases and Green area the *w*MelRio releases.

The *Wolbachia* frequency in field-caught mosquitoes increased smoothly over weeks 1–6 of *w*MelBr deployment. Surprisingly, the frequency plateaued between weeks 7–19 despite releases involving a total of 15,000 mosquitoes per week. On the last week of releases (week 20), we observed a peak of *Wolbachia* frequency of 65% (Fig 1). However, *w*MelBr frequency dropped dramatically as soon as the releases stopped. For instance, just five weeks after the releases had ended, the *Wolbachia* frequency had fallen to around 20% (Fig 1). Interestingly, *w*MelBr did not disappear altogether but rather remained relatively stable at 10–20% frequency until week 50, when new releases (with *w*MelRio) were initiated.

As soon as we observed that *Wolbachia* frequency plateaued between weeks 7–19, we conducted investigations to elucidate the reasons for such *Wolbachia* dynamics. In addition, we started doubling the numbers of mosquitoes released per week: from 7,500 in weeks 1–7 to 15,000 in weeks 13–20. However, *w*MelBr frequency still remained at around 40%.

### Quality control of *w*MelBr released mosquitoes

#### Wing size

The released mosquitoes had lower wing size variation than observed in field mosquitoes (S3A Fig). Released males were significantly larger than those collected in the field in 8 out of 20 weeks. Released females were larger than wild ones in 15 out of 20 weeks (S3B Fig).

#### Adult mortality

Adult mosquito mortality during the first 15 weeks was remarkably low, often around 5% (S3C Fig). There was no evidence of sex-biased mortality during the 20 weeks of mosquito releases.

#### Sex ratio

The sex ratio of released mosquitoes was biased towards females, except for five weeks (S3D Fig). The averaged sex ratio during the 20 weeks was 1.21:1 (F:M).

### Mitochondrial DNA (mtDNA) during *w*MelBr release

In total, 164 out of 456 *Ae*. *aegypti* tested contained the release strain mitochondrial SNP. Four of the 164 samples were not infected with *Wolbachia* (2.44%), suggesting very low level of maternal transmission leakage. These uninfected individuals were detected in samples collected on weeks 13, 14 and 26. This low level of maternal leakage is consistent with what is seen within the laboratory rearing of the release strain and indicates that *w*Mel not displayed strong transmission breakdown under field conditions despite the harsh temperatures recorded (S1 Fig).

### Bioassays with field populations and *Wolbachia* strains

**Fig 2:**
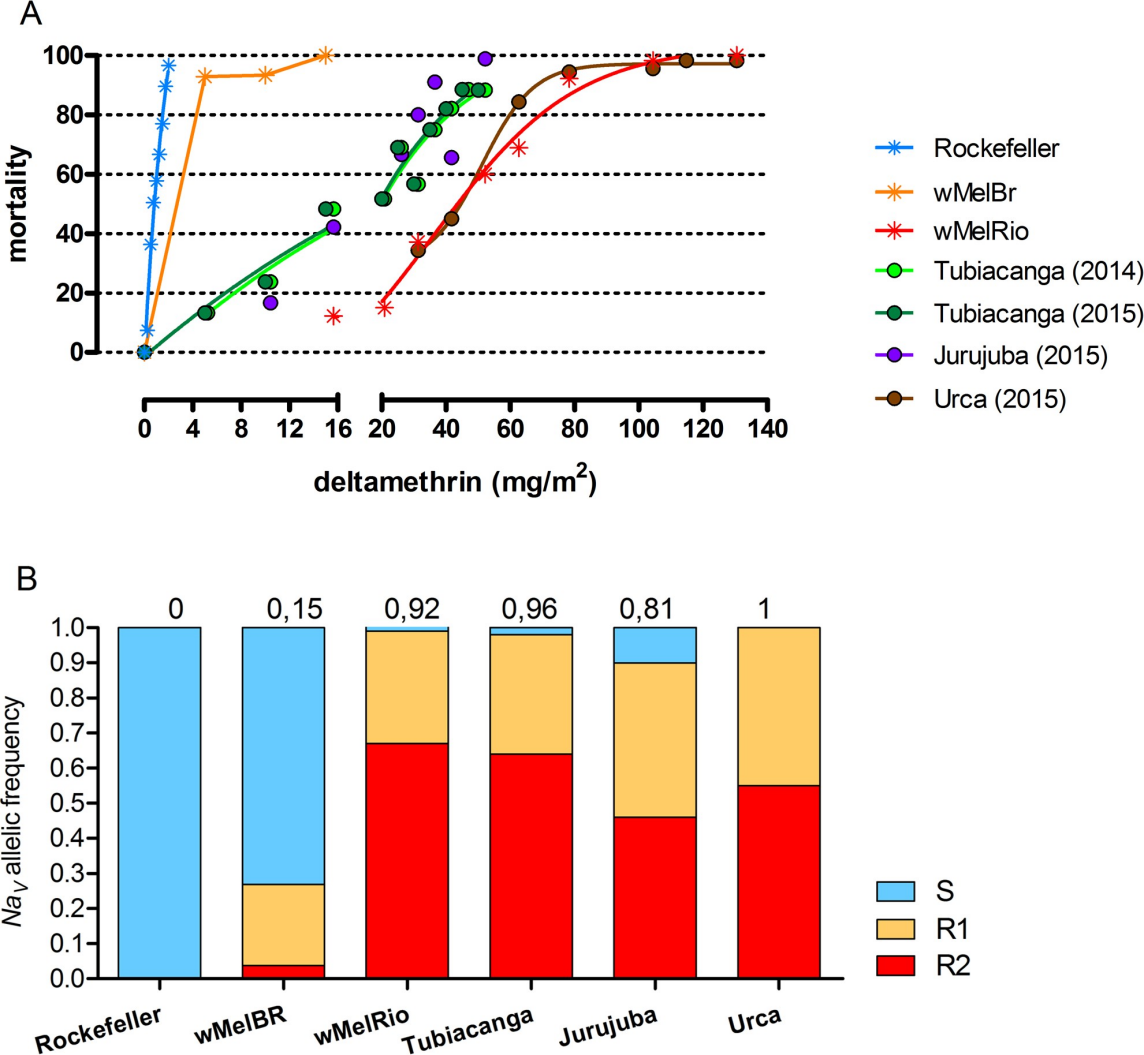
Pyrethroid resistance in two *Wolbachia*-infected strains (*w*MelBr and *w*MelRio) and three field *Aedes aegypti* populations (Tubiacanga, Jurujuba and Urca). The susceptible strain Rockefeller was used as a calibration control. A) Mortality profile of *Ae*. *aegypti* adult females exposed to the pyrethroid deltamethrin. B) Allelic frequency of population samples; numbers above bars indicate the sum of ‘resistance genotypes’ to pyrethroids, In blue Na_V_S (1016 Val^+^ + 1534 Phe^+^), in orange Na_V_R1 (1016 Val^+^ + 1534 Cys^*kdr*^) and in red Na_V_R2 (1016 Ile^*kdr*^ + 1534 Cys^*kdr*^).

The bioassays revealed a significant difference in the insecticide resistance status of *w*MelBr and *w*MelRio strains, especially for the pyrethroid deltamethrin. Considering adults, both strains were susceptible to the recently implemented pesticide malathion, but only the *w*MelRio strain could be considered as resistant to deltamethrin (pyrethroids) from the three tested wild populations (S1 Table and Fig 2A). On the other hand, individuals from the *w*MelBr population showed a similar trend to the susceptible Rockefeller strain: all individuals exposed to the test concentration of deltamethrin died (S1 Table and Fig 2A). The allelic frequency of *kdr* mutations of all tested populations reinforces the phenotypic/genotypic response of pyrethroid susceptibility of *w*MelBr and resistance of *w*MelRio, as observed in field *Ae*. *aegypti* populations (Fig 2B). In the larval assays, all tested populations were susceptible to the newly adopted diflubenzuron. However, both *w*MelBr and *w*MelRio strains were diagnosed as resistant to temephos as the field populations (RR_95_> 3,0) (S2 Table and S4 Fig).

Overall, *w*MelBr and field mosquitoes had, therefore, similar resistance levels to temephos, diflubenzuron and malathion. However, *w*MelBr was highly susceptible to deltamethrin unlike field populations. When the *w*MelRio strain was tested, comparable levels of insecticide resistance-susceptibility were found between the *w*MelRio strain and the three field populations, indicating that the *w*MelRio-released mosquitoes were as resistant as field *Ae*. *aegypti* to the chemical compounds used in the last two decades in Brazil (S1 and S2 Tables).

### Frequency of *kdr* alleles in Tubiacanga

**Fig 3:**
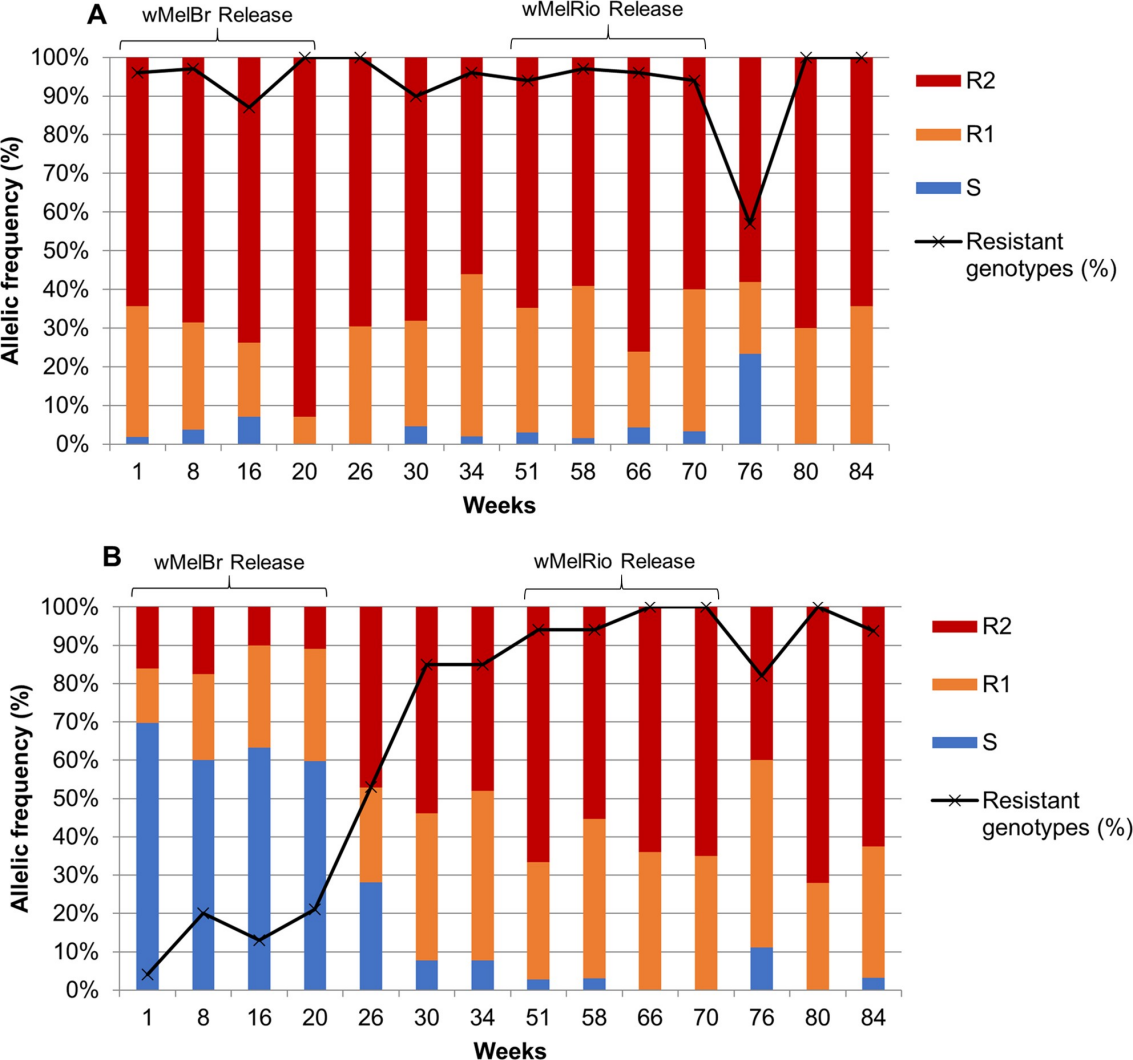
Frequency of *kdr* alleles during *Wolbachia* releases in Tubiacanga. At least 60 mosquitoes were analyzed per time point. Field-caught mosquitoes (A) without *Wolbachia*, and (B) infected with *Wolbachia*. In blue Na_V_S (1016 Val^+^ + 1534 Phe^+^), in orange Na_V_R1 (1016 Val^+^ + 1534 Cys^*kdr*^) and in red Na_V_R2 (1016 Ile^*kdr*^ + 1534 Cys^*kdr*^).

*Aedes aegypti* from Tubiacanga had a frequency of *kdr* ‘resistant genotypes’ (R1R1, R1R2 and R2R2) above 80% during the 84 weeks, which includes the periods of *Wolbachia* releases, indicating a high level of pyrethroid resistance in the field (Fig 3A). However, field-collected *w*MelBr mosquitoes retained a high frequency of the wild type allele Na_V_S, ranging from 60% to 65% up to week 20, while the ‘resistant genotypes’ did not exceed 20% in the same period (Fig 3B). Remarkably, a shift in the *kdr* allelic frequency of *Wolbachia*-infected mosquitoes was observed after *w*MelBr releases stopped, as evidenced by a continuous increase in the frequency of Na_V_R1 and Na_V_R2 alleles in weeks 26 and 30 and further stabilization up to week 84 (Fig 3B). Starting at week 30, the *kdr* allelic frequency was similar between *Ae*. *aegypti* with and without *Wolbachia*. It is of note that the allele Na_V_R2 significantly increased in wild mosquitoes during *w*MelBr releases, returning to the same level when wMelBr releases stopped, suggesting a strong selective pressure from pyrethroids in Tubiacanga (Fig 3A).

### Insecticide resistance cost in *w*MelBr

The frequencies of the R2 *kdr* alleles obtained from samples in the F1 and F18 generations were 60% and 3%, respectively. We generated uniform samples (N = 100,000) for frequencies of the homozygous genotype of the resistant allele (R2R2) within the interval 50–60% and samples of heterozygous frequencies were obtained to have a general frequency of resistant allele at 60% for F1 generation. Estimates showed a fitness cost in the F18 generation at frequencies of 3±1%, generated in a subset of 6490 values. This subset indicates a mean fitness cost of 34% (95% CI: 31–38%).

### Fitness assays of *w*MelRio strain

#### Survival

**Fig 4:**
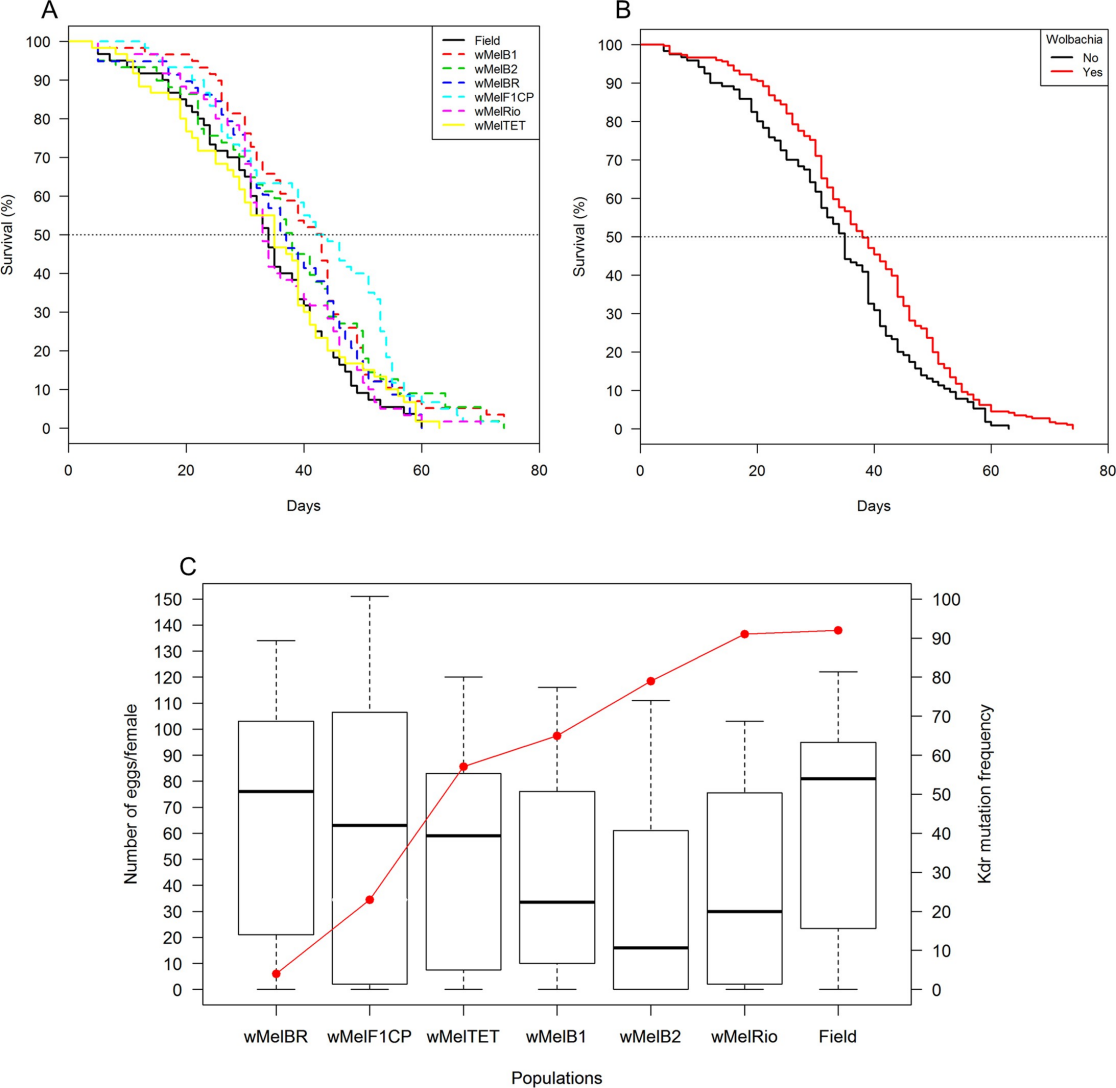
Survival curves of *Aedes aegypti* females from: (A) seven field populations from the backcrossing to produce an insecticide resistant line, (B) populations infected and uninfected with *Wolbachia*. (C) Number of eggs laid per *Aedes aegypti* female from the seven populations tested and the frequency of *kdr* mutation, represented by the red line.

Survival of mosquitoes from the second colony (*w*MelRio) was not statistically different from other samples (Fig 4A) and was strongly influenced by *Wolbachia* density (F = 9.458, d.f. = 2, p = 0.002). Namely, *Wolbachia*-infected mosquitoes presented a survivorship 38% higher when compared with their uninfected counterparts (Log-rank: χ^2^ = 8.9, d.f. = 1, P = 0.0029) (Hazard ratio: z = -2.942, ψ = 1.38, p = 0.003) (Fig 4B). Also, the frequencies of *kdr* mutation and *Wolbachia* infection, as well as the interactions among tested variables, were not informative of mosquito mortality (S3 Table). This suggests that adding insecticide resistance alleles did not affect mosquito survival.

#### Oviposition success

The proportion of *Ae*. *aegypti* females laying at least one egg varied among populations, ranging from 67.7% (B2) to 86.3% (Field). Egg laying success was strongly affected by mosquito age (χ^2^ = 58.01, d.f. = 4, P < 0.001). Female oviposition success was strongly affected by *Wolbachia* infection, with uninfected mosquitoes more likely to lay at least one egg. Oviposition success dropped more rapidly with age in mosquitoes from the populations with high *kdr* mutation frequencies (S4 Table).

#### Fecundity

We observed a slight effect of age on fecundity, with females laying fewer eggs when older in all populations tested. Eggs laid per successful females dropped from 50.5 eggs in the first clutch to 29.8 eggs in the fourth clutch. The number of eggs laid by females with *Wolbachia* dropped more rapidly than it did in uninfected populations, but bacterial density did not influence clutch size (S5 Table). Importantly, populations with higher frequencies of *kdr* mutations had a more dramatic decrease in fecundity over time (S5 Table and Fig 4C).

#### Egg hatch

**Fig 5:**
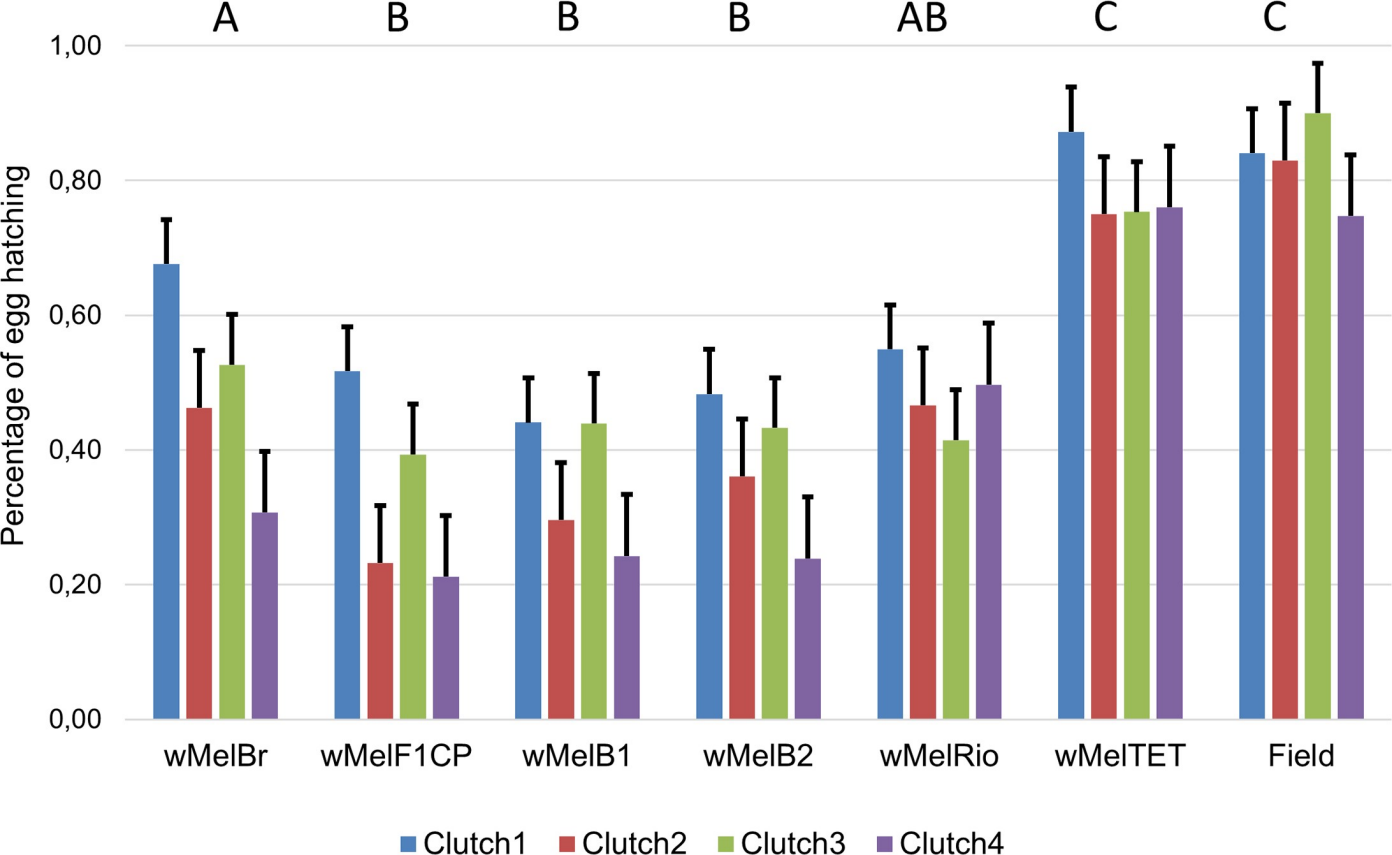
Mean and standard deviation of *Aedes aegypti* egg hatching during the first four clutches of the seven populations from the backcrossing. Different letters indicate significant differences on egg hatch averages.

A high level of variation in egg hatching was observed amongst mosquito populations, ranging from 21.1% (4^th^ clutch of *w*MelF1CP) to 89.9% (3^rd^ clutch of Field population), with higher egg hatching in the absence of the *Wolbachia* infection (Fig 5).

#### Maternal transmission

From the 249 tested mosquitoes, one female from the F1CP population was not infected with *Wolbachia*, producing an overall 99.6% rate of infection. From the 1932 individually screened larvae, 1898 (98.2%) were infected. The uninfected female of F1CP was responsible for producing 23 (67.6%) of the uninfected larvae. If excluded from analysis, *Wolbachia* maternal transmission would exceed 99.4% across the backcrossing undertaken to produce *w*MelRio (S5 Fig).

### Genetic similarities among release and field populations

After removing loci that were absent in more than 25% of individuals from each of the four samples, with minor allele frequency of 5% in at least one sample and having high log-likelihood of heterozygous genotype calls (score ≤ -10), we retained 5,118 SNPs for downstream analysis. We also removed individuals that had more than 30% of missing data across all loci, retaining 20 individuals in each of the four samples: *w*MelRio, *w*MelBr, wild (Tubiacanga, Brazil), wildAu (Gordonvale, Australia). The average depth (SE) per locus per individual was 13.5 (5.1) for *w*MelRio, 17.6 (6.4) for *w*MelBr, 10.9 (2.3) for wild, and 8.4 (2.1) for wildAu.

**Fig 6:**
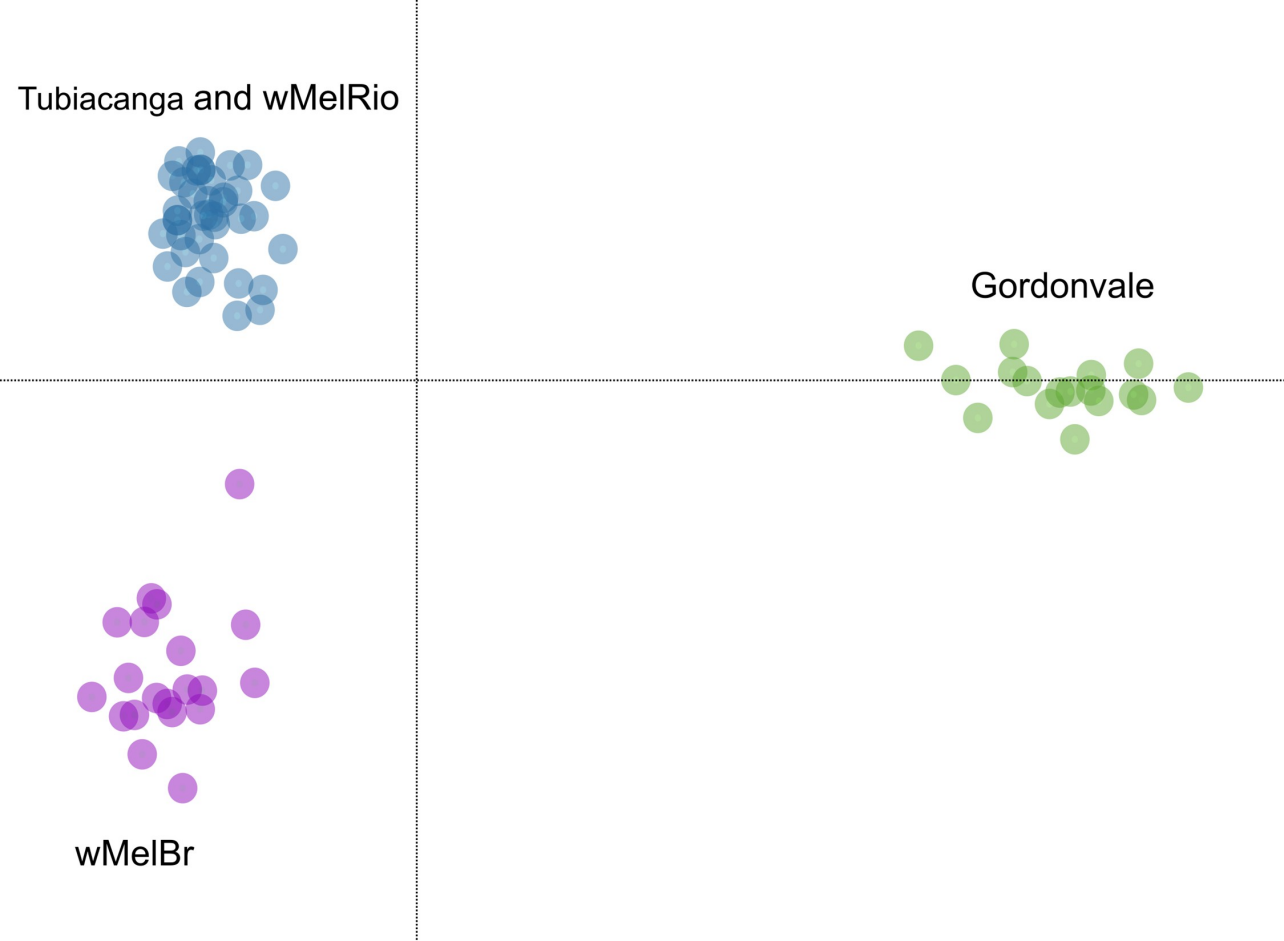
Genetic similarities and differences among *w*MelBr, *w*MelRio, Tubiacanga (Rio de Janeiro, Brazil) and Gordonvale (Cairns, Australia) *Aedes aegypti* mosquitoes as visualized by a DAPC analysis on >5000 SNPs, with data plotting samples for the two main PCs.

Analysis of genetic variation and structuring revealed that the backcrossing strategy achieved a desirable genetic composition of the *w*MelRio release material. Namely, *w*MelRio and Tubiacanga were indistinguishable in the analysis of genetic structuring. DAPC analysis showed that these two samples represent one genetic cluster that was clearly differentiated from the *w*MelBr strain and the wild Australian sample (wildAu) (Fig 6). Genome-wide *F*_ST_ (Weir and Cockerham 1984) between *w*MelRio and wild (Tubiacanga) was only 0.02, and this was significantly lower than the *F*_ST_ values between *w*MelRio and the *w*MelBr colony (0.06) or the wild Australian sample (0.114).

### *w*MelRio release in Tubiacanga

*w*MelRio releases in Tubiacanga started with a 10–20% residual frequency of *w*MelBr mosquitoes. The release period in Tubiacanga lasted 24 weeks, and *Wolbachia* frequency reached 80% on the 18^th^ week after releases started (Fig 1). One year after releases ended, the frequency of *Wolbachia* remained at 85–95%.

### Maintaining insecticide resistance in the colony during *w*MelRio releases

The frequencies of *kdr* alleles in the *w*MelRio strain were quite similar to the field population during the six months (or 10 mosquito generations) in which releases were conducted in Tubiacanga, with Na_v_R1 frequency being >30% and Na_v_R2 > 50%. The wild-type Na_v_S allele was rare, with frequencies consistently lower than 2%. The resistant genotypes (R1R1, R1R2 and R2R2) were above 90% during all releases. The same results were found for field samples during week 34 and 51 (S6 Fig).

## Discussion

The endosymbiont *Wolbachia* has been deployed in endemic areas of several countries as a strategy to reduce dengue, Zika and chikungunya transmission since mosquitoes with this bacterium have significant reduction on their vector competence to arboviruses [9–14]. We detailed *Wolbachia* invasion in Rio de Janeiro and present consistent data on the importance of profiling insecticide resistance of a native population prior to releases to maximize the likelihood of *Wolbachia* invasion. We also emphasize the importance of proper community engagement and communication, being transparent in all the processes.

Previous releases in places such as North Queensland showed a relatively constant and fast increase of *Wolbachia* frequency over weeks. For instance, an invasion of 98% was observed on Yorkeys Knob after 12 release weeks [13]. However, in Rio, invasion had plateaued at ~40–45% after the same period. Certainly, one of the major forces that may constrain *Wolbachia* invasion is the wild mosquito population size [41–42]. Tubiacanga has a greater average of mosquito/trap/day than several sites of North Queensland, but slightly smaller than in Tri Nguyen island in Vietnam where *Wolbachia* releases have also taken place. It is noteworthy that in the first weeks of releases in Rio, the ratio of wild:*w*MelBr was 1:0.5–0.7, while in North Queensland, a ratio of 1:1.2–1.7 [13,41–43]. Therefore, we initially hypothesized increasing mosquito release numbers would overcome the *w*MelBr frequency plateau. From week 13 we doubled the number of mosquitoes released, but plateau remained unaltered.

Our second hypothesis was that released mosquitoes were not fit enough to survive in the field, which was likely to be the reason why *w*MelPop strain releases failed in Tri Nguyen Island in Vietnam and North Queensland [43]. To test this, we measured mosquito wing length, adult survivorship on the first week, and sex ratio as proxies of fitness. In 15 out of 20 weeks, we released females significantly bigger than wild ones, in hope these females would not experience fitness loss due to poor rearing conditions (which was reinforced by the low mortality of adults in release cups). Finally, we checked if there were any deviation in sex ratio in release a cohort, because the ratio biased toward males would likely delay *Wolbachia* spread as it would rely more on cytoplasmic incompatibility rather than breeding by infected females. However, the sex ratio in our release cohorts was slightly biased towards females. Therefore, the evidence suggests that small wing size, low survival and male-biased sex ratio were not responsible for the plateau in infection frequency.

Another process that can hinder *Wolbachia* invasion is the loss of its perfect maternal transmission, which can be driven by the high environmental temperature fluctuations during development. Namely, larval exposure to daily fluctuating temperatures (e.g. 26–37°C or 30–40°C) during early development dramatically reduced the levels of *w*Mel in emerging *Ae*. *aegypti* females [44–46]. During *Wolbachia* deployment in Rio, Tubiacanga experienced harsh climatic conditions, with an unexpected drought summer and a constant increase in temperature starting in week 12 (S1 Fig). Despite extreme temperatures, there was no sign of a high level of incomplete maternal transmission of *Wolbachia* in Tubiacanga.

The sharp decrease of *w*MelBr-infected mosquitoes after the end of the first round of releases (Fig 1) raised concerns regarding mating abilities between *Wolbachia*-released and wild mosquitoes. Acoustic signals produced during flight play an important role in mating success, when wing-beat frequencies between male and female mosquitoes can determine females’ rejection/acceptance behaviors toward males [47–49]. Recent evidences pointed *Wolbachia* is unlikely to prevent the harmonic interaction between *Ae*. *aegypti* males and females, suggesting released individuals would meet the acoustically-related mating behavior required to promote bacteria invasion [50].

Overall, empirical data allowed us to reject the effect of (a) releasing insufficient numbers of *Wolbachia*-infected mosquitoes, (b) their reduced fitness, (c) incomplete maternal transmission to explain the plateau and the rapid decrease on *w*MelBr after the first release ended, and (d) divergent wing-beat frequency of wild mosquitoes and *Wolbachia*-infected individuals. Then, unexpectedly, the field entomology and CE teams received a clue. The owner of the local grocery store in Tubiacanga mentioned he wished releases never ended, because he was selling insecticide spray cans as never before. This led to an incident report by the CE team which provided the eureka moment for field entomology team. We then started investigating a fourth hypothesis to explain the plateau: lack of pyrethroid resistance in the released mosquitoes.

Based on evidences of the overuse of pyrethroids by local householders for personal protection, we evaluated the dynamics of *kdr* introgression, a key molecular marker for resistance to pyrethroids, during the backcrossing of wild local males to *w*Mel-infected *Ae*. *aegypti* females. The frequency of *kdr* alleles increased in every backcrossing generation and reached around 80% (60% Na_V_R2 and 20% Na_V_R1) (S7 Fig). The *knockdown* resistance is a recessive trait and the *kdr* alleles provide a fitness cost in the absence of the selective pressure of insecticide usage [28]. Thus, it is likely that the rapid decrease of *kdr* alleles in the *w*MelBr colony was a function of their high fitness cost. There are several reports of fitness cost of pyrethroid resistance on a range of relevant traits such as wing shape in *Ae*. *aegypti* [51], and reduction in male mating competitiveness in *An*. *gambiae* and *Cx*. *pipiens* [52,53]. Overall, fitness costs are expected when the resistance mechanism is related to the alterations in target site, such as *kdr* mutations. The sodium channel is a key molecule of the nervous system, its alterations generally disturb the normal physiology processes leading to several pleiotropic effects in the overall fitness. There is some evidence indicating that *Ae*. *aegypti* mosquitoes have a significant fitness cost for several life history traits due to insecticide resistance, at least under laboratory conditions [54, 55]. However, there is still little evidence pointing to costs under field conditions and how resistance genotypes fluctuate depending on insecticide use. Most importantly, here we highlight for the first time, the need to investigate the local insecticide resistance profile before *Wolbachia* deployment.

The frequency of *kdr* alleles in *Wolbachia* positive mosquitoes remained unaltered during the 20 weeks of *w*MelBr releases. The *kdr* frequency started changing on week 26 at which time all released mosquitoes were likely dead. In other words, these *kdr* frequency changes were observed in the offspring of *w*MelBr released mosquitoes. During the release period, driven by the high selection pressure of household pyrethroid spraying, a small proportion of *w*Mel-infected mosquitoes was able to survive and reproduce due to a low *kdr* frequency and this kept *Wolbachia* frequency below the threshold to promote invasion [56]. Remarkably, despite low pyrethroid resistance, *w*MelBr frequency remained at 10–15% for nine months, when the release of *w*MelRio started in Tubiacanga. The hypothesis of overuse of pyrethroids spraying by local householders is reinforced by the fluctuation of *kdr* frequencies in wild mosquitoes (Fig 3A). The frequency of Na_v_R2, the allele related with resistance and high fitness cost, increased until the releases stopped. After week 20, the pattern of genotype frequencies shifted and by week 34 it was similar to the one observed before *Wolbachia* deployment in Tubiacanga.

The main reason for failure of *w*MelBr invasion in Tubiacanga therefore seems to be related to releasing mosquitoes not fully adapted to local environmental settings. More specific, mosquitoes lacking resistance to pyrethroids. Susceptibility to pyrethroids in a site where wild population is highly resistant to this class of insecticides seems critical but is not the only factor. Adding 10% wild males every five generations to the colony intended for releases was not enough to achieve locally adapted genetic composition of the mosquitoes intended for the releases. High resistance to pyrethroids has spread across *Ae*. *aegypti* populations in Brazil [25–27,57]. Therefore, to accomplish successful *Wolbachia* deployment, countries will have to consider generating mosquitoes as resistant as the local wild population. Assessing the insecticide resistance profiles of both the wild population and *Wolbachia* mosquitoes becomes a key factor in preparation for and during the releases. *Wolbachia* was able to invade Tubiacanga when released *Ae*. *aegypti* had a genetic constitution similar to field mosquitoes. This contrasted to the situation in North Queensland, where there is no evidence for pyrethroid resistance in the field population [58].

*Wolbachia*-infected *Ae*. *aegypti* mosquitoes are currently being deployed in at least ten countries. Due to a worldwide distribution of pyrethroid-resistant alleles, many wild *Ae*. *aegypti* populations may have high levels of resistance to pyrethroids [59]. The *Ae*. *aegypti* biting rate during *Wolbachia* deployment increases due to the great influx of released mosquitoes. This disturbance may in the end lead residents to use insecticides more frequently to protect themselves, among which pyrethroids are usually the most available. It will lead to a selection of pyrethroid-resistant individuals of the natural *Ae*. *aegypti* population and kill the released *Wolbachia-*infected if they are not as resistant, precluding the success of invasion. Herein, for the first time, we empirically demonstrate that background knowledge regarding the insecticide resistance status of natural wild *Ae*. *aegypti* mosquitoes is critic to achieve *Wolbachia* usage as a disease transmission control strategy.

## Supporting information

S1 Fig Ombrothermic curve of Tubiacanga during the 20 weeks of the first *Wolbachia* release in Rio de Janeiro. Bars represent weekly rainfall, solid line the mean temperature, the dotted line represents the average maximum temperature and dashed line represent the peak temperature measured 5 km from Tubiacanga.
(TIF)

S2 Fig Schematic view of the backcrossing designed to produce an *Aedes aegypti* population resistant to pyrethroids for new releases in areas where wild mosquito population is highly resistant to insecticides (in this case Urca). (TIF)

S3 Fig Quality control of released *w*MelBr mosquitoes. (A) Wing size length of *Aedes aegypti* males (A) and females (B) released in the 20 weeks of *Wolbachia* deployment in Tubiacanga. Each week had 30 individuals randomly selected. The asterisk shows significance when released mosquitoes had wing length significantly bigger than wild-caught ones. (C) Mean and confidence interval of the percentage of dead *Aedes aegypti* mosquitoes after release cups went to the field and back to the insectary. (D) Mean and confidence interval of sex ratio (female:male) of released mosquitoes. Points above the dotted line indicate sex ratio biased towards females. The red dotted line indicates the average sex ratio during releases.
(TIF)

S4 Fig Dose x mortality profile in *Aedes aegypti* adult females exposed to a gradient of concentrations of the larvicide Temephos for one hour, with mortality scored 24h later. (TIF)

S5 Fig Frequency of *Wolbachia*-positive offspring during the first and fourth clutches of their relatives. Data gathered from 1932 individually screened larvae.
(TIF)

S6 Fig The frequencies of Na_v_R1 and Na_v_R2 alleles and genotypic frequency in *w*MelRio strain colony maintained under lab conditions during the second release in Tubiacanga. The two columns on the right represent the frequency of *kdr* alleles in the field population, week 34 representing the period between *w*MelBr and *w*MelRio releases, and week 51 during the *w*MelRio release. In blue Na_V_S (1016 Val^+^ + 1534 Phe^+^), in orange Na_V_R1 (1016 Val^+^ + 1534 Cys^*kdr*^) and in red Na_V_R2 (1016 Ile^*kdr*^ + 1534 Cys^*kdr*^).
(TIF)

S7 Fig Allelic frequency of the susceptible wild-type (Na_v_S), the *kdr* allele with a substitution restricted to the 1534 position (Na_v_^R1^), or concurrent substitutions in both 1534 and 1016 sites (Na_v_^R2^) during backcrossing to produce the strain wMelBr. (TIF)

S1 Table Profile of wMelRio and three Ae. aegypti mosquitoes from local field populations (Tubiacanga, Jurujuba and Urca) exposed to two adulticides: (A) the organophosphate malathion (mg/m^2^) and (B) the pyrethroid deltamethrin (mg/m^2^) with an exposure of 120 minutes. The dose used to evaluate the resistance ratio of mosquito populations to both insecticides killed all the individuals from the wMelBr and Rock populations.
(DOCX)

S2 Table Profile of wMelBr, wMelRio and three Ae. aegypti mosquitoes from local field populations (Tubiacanga, Jurujuba and Urca) exposed to two larvicides: (A) diflubenzuron (μg/L) and (B) the organophosphate temephos (mg/mL). Diflubenzuron is currently employed by the Brazilian Ministry of Health.
(DOCX)

S3 Table Analysis of variance of the influence of *kdr* frequency, *Wolbachia* presence and density on the survival of *Aedes aegypti* females. (DOCX)

S4 Table Logistic regression analysis of the influence of mosquito age, *kdr* frequency, *Wolbachia* presence and density on laying at least one egg during the first five clutches. (DOCX)

S5 Table Repeated measures analysis (with clutch size as the repeatedly measured variable) of the square-root of the number of eggs laid by successful *Aedes aegypti* females in the first five oviposition cycles. (DOCX)
