# Supplementary figures and images for: Matching the genetics of released and local Aedes aegypti populations is critical to assure Wolbachia invasion
Source: PLoS Negl Trop Dis. 2019 Jan 8;13(1):e0007023. doi: 10.1371/journal.pntd.0007023 (PMC6338382; doi:10.1371/journal.pntd.0007023)

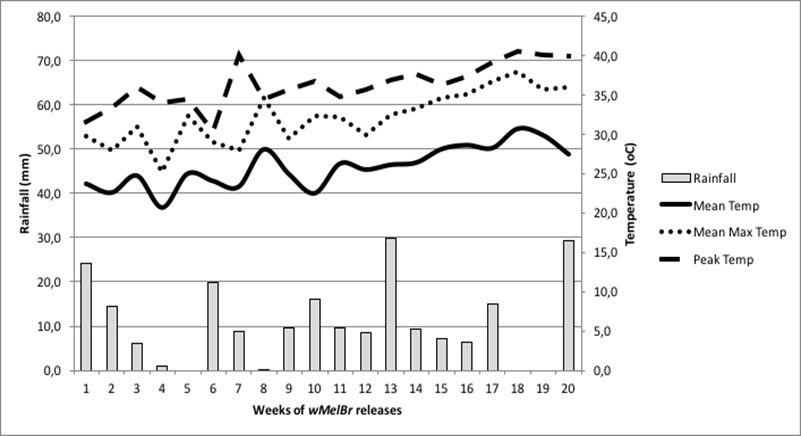

Supplement: S1 Fig — Bars represent weekly rainfall, solid line the mean temperature, the dotted line represents the average maximum temperature and dashed line represent the peak temperature measured 5 km from Tubiacanga. (TIF) [file pntd.0007023.s001.tif]

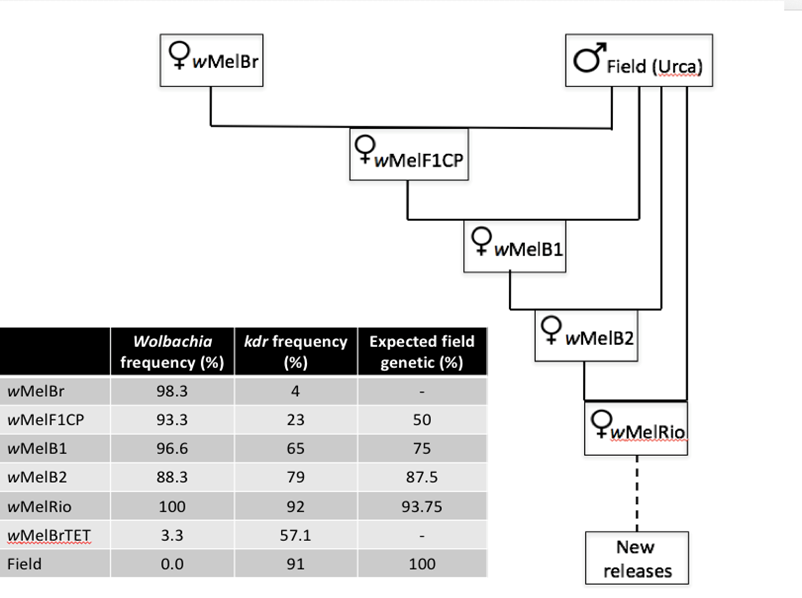

Supplement: S2 Fig — (TIF) [file pntd.0007023.s002.tif]

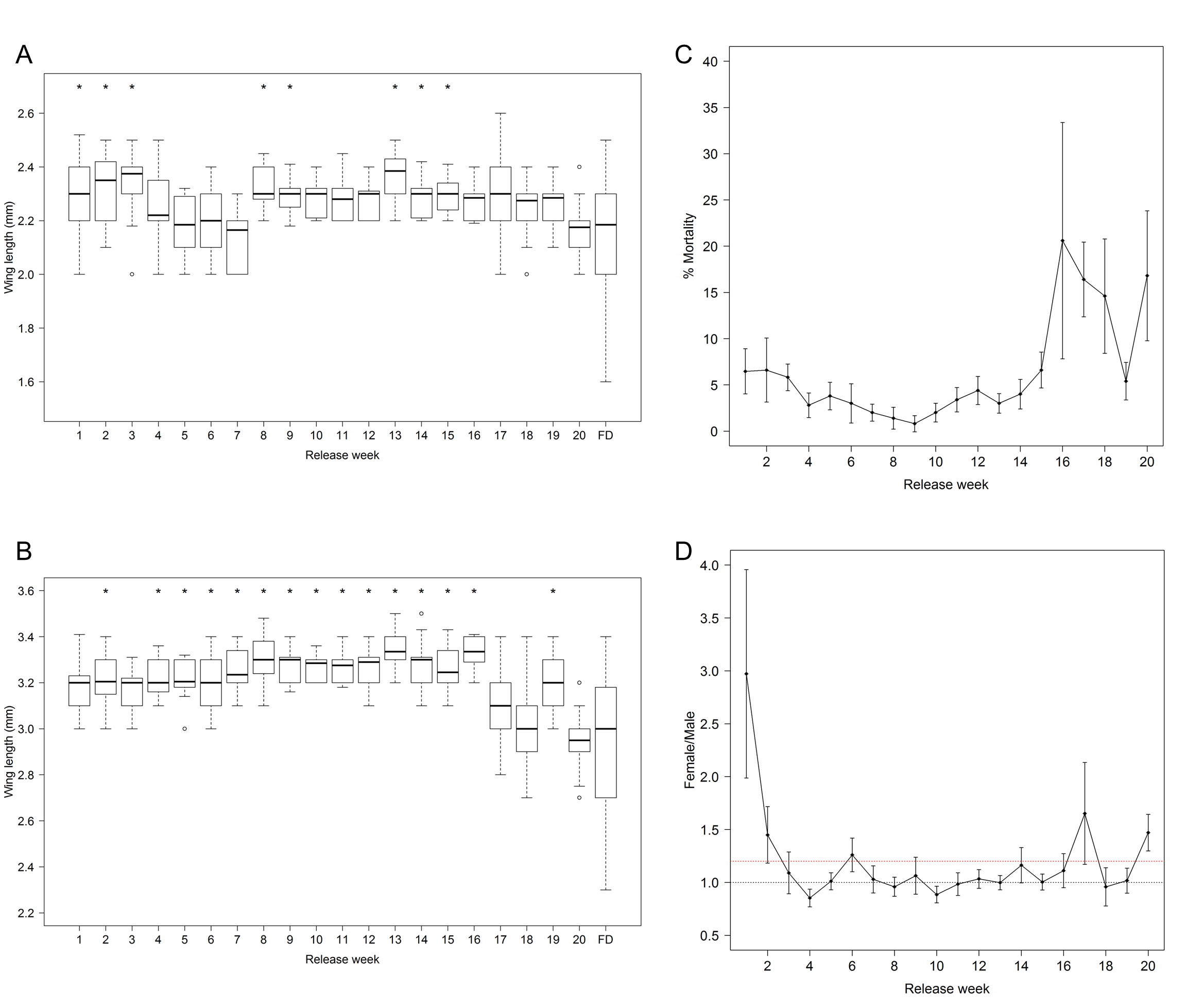

Supplement: S3 Fig — (A) Wing size length of Aedes aegypti males (A) and females (B) released in the 20 weeks of Wolbachia deployment in Tubiacanga. Each week had 30 individuals randomly selected. The asterisk shows significance when released mosquitoes had wing length significantly bigger than wild-caught ones. (C) Mean and confidence interval of the percentage of dead Aedes aegypti mosquitoes after release cups went to the field and back to the insectary. (D) Mean and confidence interval of sex ratio (female:male) of released mosquitoes. Points above the dotted line indicate sex ratio biased towards females. The red dotted line indicates the average sex ratio during releases. (TIF) [file pntd.0007023.s003.tif]

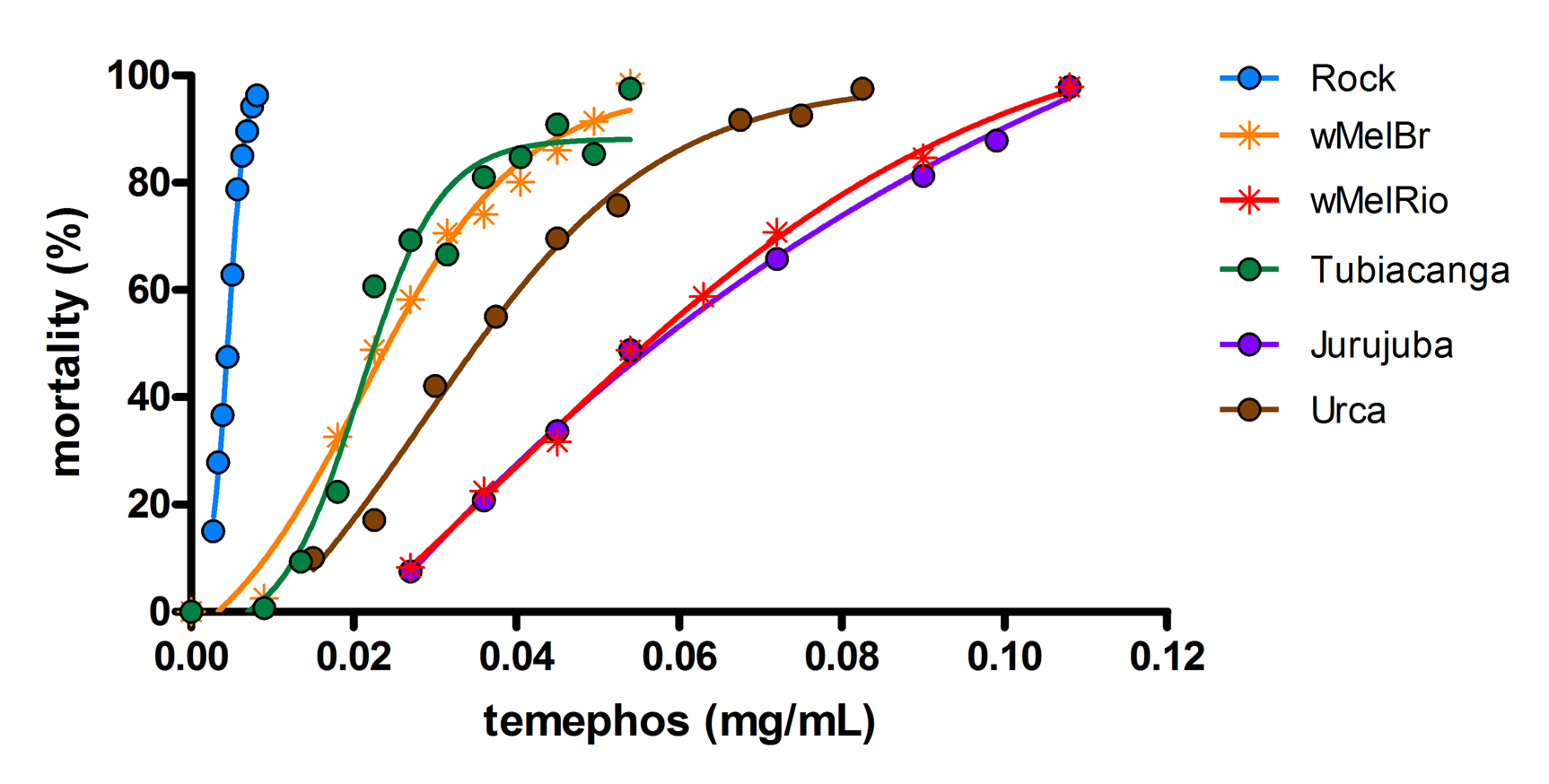

Supplement: S4 Fig — (TIF) [file pntd.0007023.s004.tif]

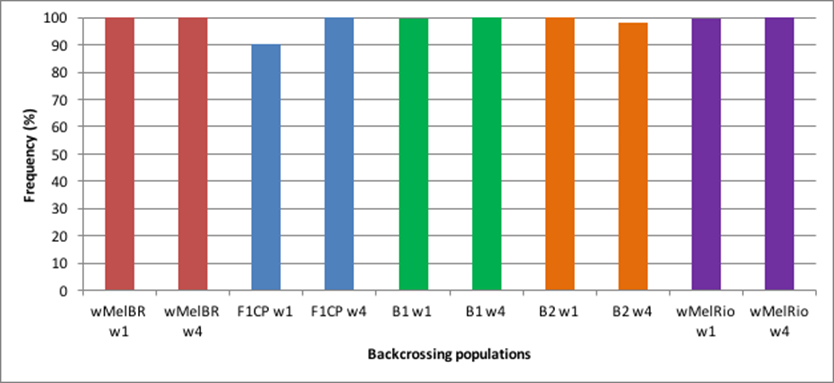

Supplement: S5 Fig — Data gathered from 1932 individually screened larvae. (TIF) [file pntd.0007023.s005.tif]

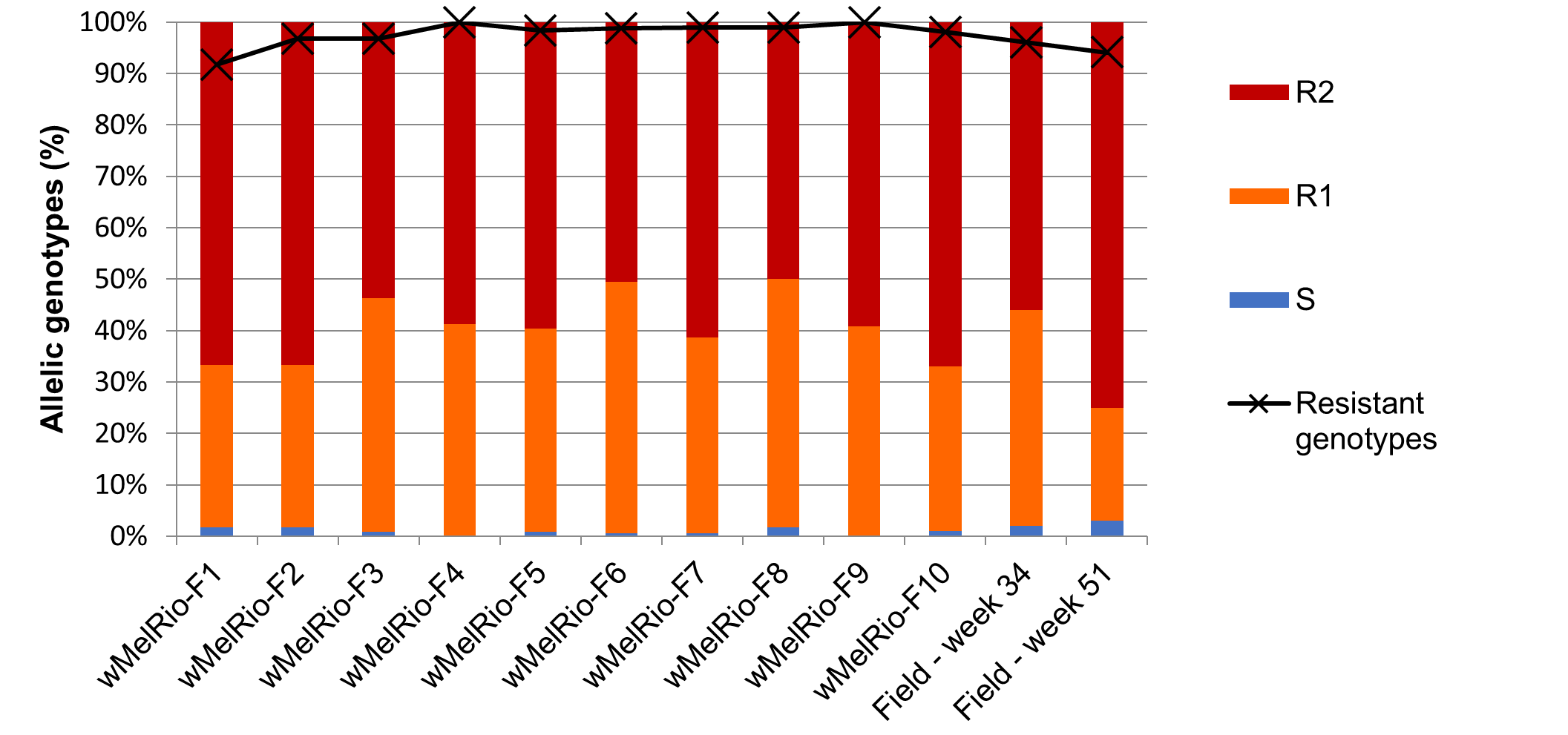

Supplement: S6 Fig — The two columns on the right represent the frequency of kdr alleles in the field population, week 34 representing the period between wMelBr and wMelRio releases, and week 51 during the wMelRio release. In blue NaVS (1016 Val+ + 1534 Phe+), in orange NaVR1 (1016 Val+ + 1534 Cyskdr) and in red NaVR2 (1016 Ilekdr + 1534 Cyskdr). (TIF) [file pntd.0007023.s006.tif]

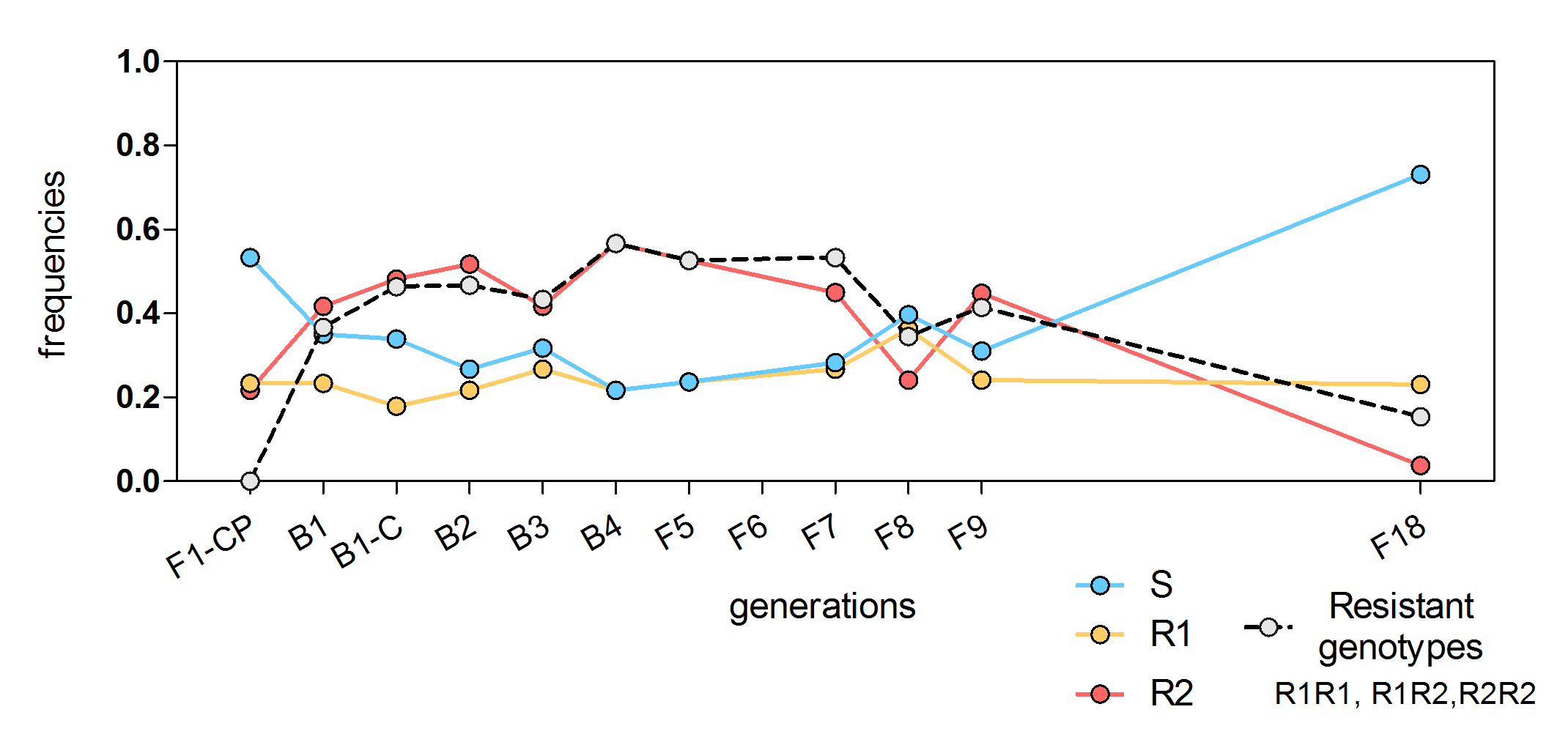

Supplement: S7 Fig — (TIF) [file pntd.0007023.s007.tif]
