# Supplementary material for: Matching the genetics of released and local Aedes aegypti populations is critical to assure Wolbachia invasion
Source: PLoS Negl Trop Dis. 2019 Jan 8;13(1):e0007023. doi: 10.1371/journal.pntd.0007023 (PMC6338382; doi:10.1371/journal.pntd.0007023)
Supplement: S1 Table — Profile of wMelRio and three Ae. aegypti mosquitoes from local field populations (Tubiacanga, Jurujuba and Urca) exposed to two adulticides: (A) the organophosphate malathion (mg/m2) and (B) the pyrethroid deltamethrin (mg/m2) with an exposure of 120 minutes. The dose used to evaluate the resistance ratio of mosquito populations to both insecticides killed all the individuals from the wMelBr and Rock populations. (DOCX) [file pntd.0007023.s008.docx]

(A)

| Malathion | | | | | | | | | | |
| --- | --- | --- | --- | --- | --- | --- | --- | --- | --- | --- |
| Lineage/ populations | slope |  | LC_50_ (IC_95_) | | RR_50_ |  | LC_90_ (IC_95_) | | RR_90_ |  |
| Rockefeller | 8.606 |  | 0.052 | (0.026-0.105) | - |  | 0.149 | (0.066-0.337) | - |  |
| wMelRio | 5.127 |  | 0.202 | (0.171-0.239) | 3,85 |  | 0.359 | (0.279-0.463) | 2,40 |  |
| Jurujuba | 6.320 |  | 0.197 | (0.179-0.217) | 3,75 |  | 0.314 | (0.276-0.359) | 2,10 |  |
| Urca | 6.322 |  | 0.198 | (0.187-0.206) | 3,76 |  | 0.315 | (0.301-0.332) | 2,10 |  |

(B)

| Deltamethrin | | | | | | | | | | |
| --- | --- | --- | --- | --- | --- | --- | --- | --- | --- | --- |
| Lineage/ populations | slope |  | LC_50_ (IC_95_) | | RR_50_ |  | LC_90_ (IC_95_) | | RR_90_ |  |
| Rockefeller | 4.953 |  | 1.051 | (0.96-1.16) | - |  | 1.915 | (1.65-2.22) | - |  |
| wMelRio | 3.591 |  | 38.842 | (32.79-45.98) | 37.0 |  | 88.310 | (67.54-115.90) | 46.2 |  |
| Tubiacanga | 2.048 |  | 17.931 | (12.7-25.3) | 17.1 |  | 62.602 | (36.2-108.3) | 32.8 |  |
| Jurujuba | 4.186 |  | 18.277 | (16.35-20.35) | 17.4 |  | 37.005 | (32.55-42.32) | 19.4 |  |
| Urca | 4.566 |  | 40.123 | (35.57-45.05) | 38.2 |  | 76.424 | (67.32-87.28) | 40.0 |  |
