# Supplementary material for: Matching the genetics of released and local Aedes aegypti populations is critical to assure Wolbachia invasion
Source: PLoS Negl Trop Dis. 2019 Jan 8;13(1):e0007023. doi: 10.1371/journal.pntd.0007023 (PMC6338382; doi:10.1371/journal.pntd.0007023)
Supplement: S2 Table — Profile of wMelBr, wMelRio and three Ae. aegypti mosquitoes from local field populations (Tubiacanga, Jurujuba and Urca) exposed to two larvicides: (A) diflubenzuron (μg/L) and (B) the organophosphate temephos (mg/mL). Diflubenzuron is currently employed by the Brazilian Ministry of Health. (DOCX) [file pntd.0007023.s009.docx]

(A)

| Diflubenzuron | | | | | | | | | |
| --- | --- | --- | --- | --- | --- | --- | --- | --- | --- |
| Lineage/ populations | slope |  | LC_50_ (IC_95_) | | RR_50_ |  | LC_90_ (IC_95_) | | RR_90_ |
| Rockefeller | 5.152 |  | 0.900 | (0.415-1.951) | - |  | 2.288 | (1.001-5.229) | - |
| wMelBr | 5.204 |  | 0.794 | (0.243-2.594) | 0.882 |  | 3.309 | (0.543-20.167) | 1.4 |
| Tubiacanga | 4.362 |  | 1.378 | (0.682-2.787) | 1.531 |  | 2.600 | (1.520-4.449) | 1.1 |

(B)

| Temephos | | | | | | | | | | | | |
| --- | --- | --- | --- | --- | --- | --- | --- | --- | --- | --- | --- | --- |
| Lineage/ populations | Slope |  | LC_50_ (IC_95_) | | RR_50_ |  | | LC_90_ (IC_95_) | | | RR_90_ |  |
| Rockefeller | 5.962 |  | 0.0043 | (0.0042-0.0044) | - | |  | | 0.0071 | (0.0068-0.0073) |  |  |
| *w*MelRio | 4.919 |  | 0.0541 | (0.0510-0.0575) | 12.61 | |  | | 0.0986 | (0.0887-0.1103) | 14.0 |  |
| *w*MelBr | 12.528 |  | 0.0239 | (0.0157-0.0363) | 5.62 | |  | | 0.0451 | (0.0275-0.0738) | 6.4 |  |
| Tubiacanga | 13.242 |  | 0.0255 | (0.0176-0.0370) | 6.05 | |  | | 0,0452 | (0,0285-0,0716) | 6.4 |  |
| Jurujuba | 4.760 |  | 0.0550 | (0.0513-0.0590) | 18.85 | |  | | 0.1022 | (0.0914-0.1148) | 14.5 |  |
| Urca | 4.303 |  | 0.0341 | (0.0319-0.0363) | 7.92 | |  | | 0.0676 | (0.0617-0.0744) | 9.6 |  |
