# Supplementary material for: Matching the genetics of released and local Aedes aegypti populations is critical to assure Wolbachia invasion
Source: PLoS Negl Trop Dis. 2019 Jan 8;13(1):e0007023. doi: 10.1371/journal.pntd.0007023 (PMC6338382; doi:10.1371/journal.pntd.0007023)
Supplement: S3 Table — (DOCX) [file pntd.0007023.s010.docx]

| Source | d.f. | Sum of squares | F | p |
| --- | --- | --- | --- | --- |
| *kdr* | 1 | 47.70 | 0.319 | 0.572 |
| *Wolbachia* density | 1 | 1413.75 | 9.458 | **0.002** |
| *Wolbachia* infection | 1 | 132.99 | 0.889 | 0.346 |
| *kdr* AND *Wolbachia* infection | 1 | 21.24 | 0.142 | 0.706 |
| *kdr* AND *Wolbachia* density | 1 | 4.42 | 0.029 | 0.863 |
