# Supplementary material for: Matching the genetics of released and local Aedes aegypti populations is critical to assure Wolbachia invasion
Source: PLoS Negl Trop Dis. 2019 Jan 8;13(1):e0007023. doi: 10.1371/journal.pntd.0007023 (PMC6338382; doi:10.1371/journal.pntd.0007023)
Supplement: S4 Table — (DOCX) [file pntd.0007023.s011.docx]

| Source | d.f. | χ^2^ | *P-value* |
| --- | --- | --- | --- |
| Age | 4 | 58.01 | **<0.001** |
| *Wolbachia* infection | 1 | 4.07 | **0.043** |
| *Kdr* | 1 | 4.04 | **0.044** |
| *Wolbachia* density | 1 | 0.001 | 0.972 |
| *Wolbachia* infection AND *kdr* | 1 | 0.57 | 0.451 |
| *Wolbachia* density AND *kdr* | 1 | 0.46 | 0.497 |
| Age AND *Wolbachia* infection | 4 | 3.97 | 0.408 |
| Age AND *kdr* | 4 | 8.62 | 0.071 |
| Age AND *Wolbachia* density | 4 | 1.16 | 0.883 |
