# Supplementary material for: Matching the genetics of released and local Aedes aegypti populations is critical to assure Wolbachia invasion
Source: PLoS Negl Trop Dis. 2019 Jan 8;13(1):e0007023. doi: 10.1371/journal.pntd.0007023 (PMC6338382; doi:10.1371/journal.pntd.0007023)
Supplement: S5 Table — (DOCX) [file pntd.0007023.s012.docx]

| Source | Numerator d.f. | Denominator d.f. | *F* | *P* |
| --- | --- | --- | --- | --- |
| Clutch AND *kdr* | 1 | 214 | 7.47 | **<0.001** |
| Clutch AND *Wolbachia* density | 1 | 214 | 0.39 | 0.818 |
| Clutch AND *Wolbachia* infection | 1 | 214 | 4.15 | **0.003** |
